# BioJazz: *in silico* evolution of cellular networks with unbounded complexity using rule-based modeling

**DOI:** 10.1093/nar/gkv595

**Published:** 2015-06-22

**Authors:** Song Feng, Julien F. Ollivier, Peter S. Swain, Orkun S. Soyer

**Affiliations:** 1 School of Life Sciences, University of Warwick, Coventry, United Kingdom; 2 Department of Physiology, McGill University, Montreal, Quebec; 3 SynthSys, The University of Edinburgh, Edinburgh, United Kingdom

## Abstract

Systems biologists aim to decipher the structure and dynamics of signaling and regulatory networks underpinning cellular responses; synthetic biologists can use this insight to alter existing networks or engineer *de novo* ones. Both tasks will benefit from an understanding of which structural and dynamic features of networks can emerge from evolutionary processes, through which intermediary steps these arise, and whether they embody general design principles. As natural evolution at the level of network dynamics is difficult to study, *in silico* evolution of network models can provide important insights. However, current tools used for *in silico* evolution of network dynamics are limited to *ad hoc* computer simulations and models. Here we introduce BioJazz, an extendable, user-friendly tool for simulating the evolution of dynamic biochemical networks. Unlike previous tools for *in silico* evolution, BioJazz allows for the evolution of cellular networks with unbounded complexity by combining rule-based modeling with an encoding of networks that is akin to a genome. We show that BioJazz can be used to implement biologically realistic selective pressures and allows exploration of the space of network architectures and dynamics that implement prescribed physiological functions. BioJazz is provided as an open-source tool to facilitate its further development and use. Source code and user manuals are available at: http://oss-lab.github.io/biojazz and http://osslab.lifesci.warwick.ac.uk/BioJazz.aspx.

## INTRODUCTION

Cellular networks allow organisms to sense and process environmental information and thereby implement phenotypic behaviors that enable survival. Hence, it is of fundamental interest to understand their structure and dynamics either by experimental and modeling studies on specific examples (1–3) or by searching for recurring structural motifs in large classes of systems (4–7). Collectively, these approaches have identified key dynamical features, such as ultrasensitivity and bistability (8), and elucidated biochemical elements used for their implementation, such as feedback loops, scaffold proteins and phosphorylation cycles (9–14). Despite these insights, however, we still lack an understanding of the evolutionary origins of the dynamical and structural features of such networks, limiting our ability to make functional predictions based solely on the presence or absence of these features (15). Furthermore, network elements identified from current day organisms might not constitute the only feasible solutions for achieving a specific physiological task or implementation of a specific dynamical feature. The understanding of the ‘possible solution space’ is thus mostly lacking, but could be essential from the perspective of engineering biological systems through synthetic biology (16).

One approach for understanding the evolutionary processes leading to current day network elements and for exploring the space of possible solutions is to re-create the evolutionary dynamics of cellular networks *in silico*. This task requires computational tools that are intuitive to use, yet are sufficiently complex to capture the system dynamics of known cellular networks. Modeling of the evolution of cellular networks has so far been attempted either for exploiting evolution as a design tool (e.g. (16,17)) or for interrogating evolutionary pressures leading to particular network properties (e.g. (18–20)). It is desirable to develop further general computational tools that can achieve both aims and that can allow unconstrained modeling of evolution, while maintaining a realistic representation of biochemistry and system dynamics. Most previous studies either focused on modeling of evolution of large networks without incorporating dynamics (21–28) or explicitly considered temporal dynamics of the systems that are being evolved (using for example ordinary differential equations; ODEs) (e.g. (29–34)) with bounds restricting the size and complexity of the reaction networks. When the modeling of dynamics is combined with unbounded system size as has been done in the study of evolution of gene networks through duplication (35), it was possible to better understand the evolutionary solution space. In addition, each of the different models of cellular network evolution addresses specific aspects of biology (e.g. the role of duplication in the evolution of robustness), but there are still some biomolecular aspects not yet incorporated in evolutionary models of cellular networks. A particular example is the allosteric and domain-based nature of proteins, which is shown to be relevant for the system dynamics in the context of signaling networks (36,37).

Here, we introduce an extendable, general tool that provides biologically realistic simulation of the evolution of dynamic biochemical networks. The tool, called BioJazz, combines a rule-based modeling approach (38–40) with evolutionary simulation, allowing for evolution of cellular systems without any need for *a priori* limitations on the systems that can evolve. Thus, what is meant here by ‘without limitations’ is that the structure, size and complexity of the system that is taken as an evolving entity (i.e. the modeled cellular system) is not bounded in any way (other than computational limitations). Rule-based modeling is perfectly suited for this evolutionary approach, as it is developed in the first place to overcome the combinatorial complexity arising from accounting for all possible interactions in a given biological system (40,41). The rule-based modeling approach and the genome-like encoding of the network we adopt also allow biologically realistic mutational events to be modeled naturally. BioJazz has the ability to change and evolve networks with respect to both topology and biochemical parameters, by starting either from a designed network *de novo* or from a partially or completely functional seed network.

We demonstrate the use of Biojazz by examining the evolution of network dynamics for two sample cases, demonstrating evolution of network architectures for ultrasensitive and adaptive response dynamics. We also use these examples to demonstrate the effects of the parameters of the simulation algorithm on the performance and evolutionary space of such signaling networks.

## MATERIALS AND METHODS

### Representing network interactions: rule-based model

Previous attempts to model the evolution of cellular networks relied on *ad hoc* approaches to encode network architecture and dynamics (e.g. see (16–20)). Here, we make use of recently developed rule-based approaches to enable a flexible encoding of cellular networks, allowing for both realistic representation of their biochemistry and for *in silico* evolution with unbounded complexity. Rule-based approaches are developed for addressing the combinatorial complexity arising in even biologically simple reaction systems (40,41) and, hence, are well suited to be combined with an evolutionary approach. Although several rule-based methods are now available (38,39,42–44), we choose to use the Allosteric Network Compiler (ANC) (38), because it systematically incorporates the allosteric and modular nature of proteins (note that the software structure of BioJazz allows other rule-based models to be incorporated in subsequent developments). ANC is a stand-alone, rule-based compiler, which turns a high-level description of allosteric proteins into the corresponding set of biochemical equations.

ANC has been described previously (38). In brief, it models proteins as multi-domain entities, where each domain is an allosteric unit that can adopt two general conformational states following the Monod-Wyman-Changeux (MWC) allosteric model (45). The two conformational states of each domain can be described as relaxed, ‘R’, and tense, ‘T’, and are assumed to have distinct free energies as well as different binding and enzymatic characteristics. Indeed, the binding and catalytic activity of reactive sites within a domain are dependent on, and only on, the conformational state of that domain. Biochemically, domains are independent sub-units of a protein, comprising reactive sites such as catalytic or post-translational modification sites (as explained below). This choice is inspired by the structure and function of multi-domain proteins in nature. In most cases, signaling proteins are functionally modular and make use of distributed surface docking sites for recognition (46), which has been demonstrated in both natural (47,48) and synthetic protein circuits (37,49–52). For example, the protein family of transcription factor-IIIA (TFIIIA) contains proteins that have three linked folded domains that are involved in binding and are regulated independently of each other (53).

ANC implements allosteric regulation by modeling the effect of any binding event or post-translational modification on a given domain through modifying the R-T transition dynamics of that domain. Thus, other molecules binding to a given protein can be seen as ‘modifiers’, which alter the distribution of the R and T states of the domain that they bind. The transition between the R and T states is governed by the free energies of these states as well as any transition state between them (see Supplementary Data). ANC can thereby model a cellular network as a given set of proteins that comprise domains and that interact through binding and through covalent modifications of reactive sites on those domains. Any domain can be allosteric, in which case, it would have distinct R and T states with associated allosteric rate constants. Any modifications would result in altering the dynamics of the R-T transitions. As explained further below, in BioJazz's application of ANC, the $k_{RT}$, $k_{TR}$, and ${\rm \Phi }$ values for each domain and the ${\rm \Gamma }_i$ values for different reactive sites on a given domain are free to evolve. Note that this freedom allows us to implement easily and naturally the evolution of both individual proteins with domains that have specific internal dynamics and protein interaction networks, via the definition of binding specificities among reactive sites and ${\rm \Gamma }$ parameters.

### Encoding network information: a binary string as a synthetic genome

**Figure 1.**
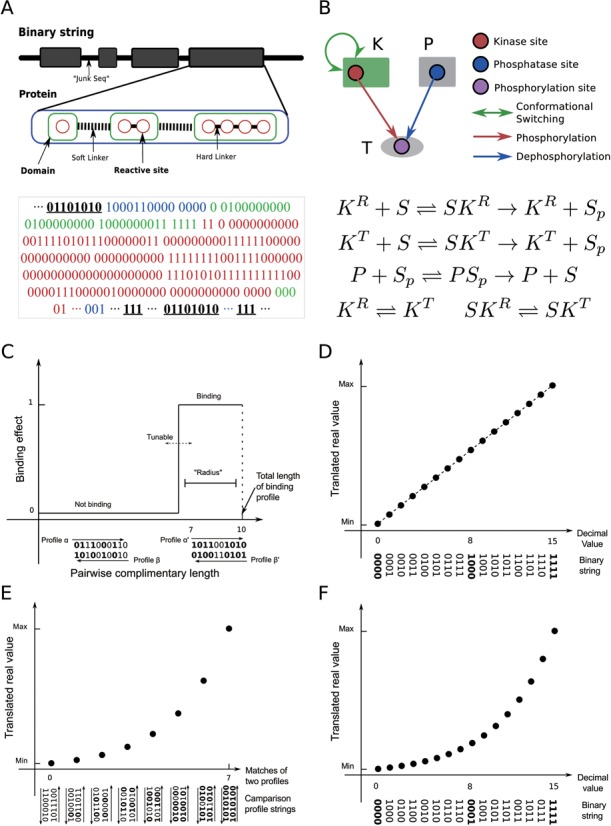
The ‘Genome’ structure and scaling method used to encode cellular networks. (**A**) A cartoon representation of the binary string encoding the information needed to build an ANC model. The string has a hierarchical structure explained in the main text. (**B**) A cartoon representation and the resulting biochemical reactions of a sample reaction network that can be derived from a binary string (as shown in panel A). (**C**) Determination of binding between two reactive sites from a binary string segment (‘Materials and Methods’ section). The y-axis shows the binding effect; the x-axis shows complementary matches between two binding profiles. The threshold for binding, determining protein promiscuity, is user-defined. (**D** and **F**) Scaling of the binary string encoding of parameters into real values using a linear scaling. The y-axis corresponds to parameter values; the x-axis shows decimal values of the binary string. For different parameters (i.e. , protein concentrations, and rate constants of the conformational transition), a linear (D) or logarithmic (F) scaling is used (‘Materials and Methods’ section). (**E**) The scaling of the binary string encoding of parameters relating to binding-mediated reactions. The y-axis corresponds to a kinetic rate value; the, x-axis shows a comparison between strings encoding for binding parameters of two reactive sites as explained in ‘Materials and Methods’ section.

By describing the interaction rules as well as their allosteric effects, ANC allows modeling of a reaction network of arbitrary size and complexity. To evolve cellular networks *in silico*, one needs a method to store and mutate the corresponding protein interaction rules and parameters. In BioJazz, we encode the information in an ANC model as a binary string (Figure 1A). Using a set of ‘translation’ rules, all the information required to build an ANC model (Figure 1B) can then be extracted from a given string (Figure 1C–F).

The structure of the binary string is similar to a natural genome, where ‘non-coding’ sections separate sections encoding information. This division is implemented by using ‘start’ and ‘stop’ strings and allows an increase in evolutionary innovations through mutations (see below). We can also start evolutionary simulations from entirely random initial points (i.e. a randomly generated binary string). The coding sections of the binary string encode the structure, dynamics and interactions of proteins as explained in detail below and in Table 1. Thus, we can parse a given binary string and ‘translate’ into an ANC model (Figure 1B).

**Table 1.** Details of structural and ANC encodings implemented in the binary string

Field name	Length (L)	RegExp	Description
*Binary String*			
PRE JUNK	Any	[01]*	Zero or more bits representing untranslated sequence preceding first protein
Protein	L{protein}	[protein]+	One or more proteins separated by untranslated sub-sequences
POST JUNK	Any	[01]*	Zero or more bits representing untranslated sequence following last protein
*Protein*			
START CODE	8	01111110	Fixed pattern before the string of protein indicating the starting point of a protein
Concentration	10	[01]L	Loglinear scaled, encodes initial concentration of protein
UNUSED	4	[01]L	Reserved field
Domain	L{domains}	[domain]+	One or more domains separated by a soft linker pattern ‘001’
STOP CODE	3	111	Terminate the protein
*Domain*			
Allosteric flag	1	[01]L	Determine the domain is allosteric regulated or not
R ↔ T transition rate	10	[01]L	Loglinear scaled, kinetic parameter of conformation transition in basal level.
Φ	10	[01]L	Linear scaled into [0,1], determines changes in allosteric equilibrium under interactions.
UNUSED	4	[01]L	
Reactive sites	L{sites}	[site]+	One or more reactive sites separated by a hard linker pattern ‘000’
*Protodomain*			
Type	2	[01]L	Reactive site type, 00 = bsite, 01 = msite, 11 = csite
Substrate polarity	1	[01]L	A csite to modify (0) or unmodify (1) the substrate
Binding profile	10	[01]L	Determines ligands pairs with sufficiently complementary strings
$k_f$ profile	20	[01]L	Loglinear scaled, determines association kinetics with Hamming distance from pairing reactive sites
$k_b$ profile	20	[01]L	Loglinear scaled, determining disassociation kinetics with Hamming distance from pairing reactive sites
$k_p$ profile	10	[01]L	Loglinear scaled, for csite only, determines rate of post-translational modification
$k_{eq}$ ratio	10	[01]L	Loglinear scaled, determines allosteric effect of msite modification, see $\Gamma$ in ANC model
$k_f$ polarity mask	20	[01]L	XOR with $k_{f}$ profile to determine profile of modified reactive site (msite = 1)
$k_b$ polarity mask	20	[01]L	XOR with $k_{b}$ profile to determine profile of modified reactive site (msite = 1)
$k_f$ conformation mask	20	[01]L	XOR with $k_{f}$ profile to determine new profile of T conformation
$k_b$ conformation mask	20	[01]L	XOR with $k_{b}$ profile to determine new profile of T conformation
$k_p$ conformation mask	20	[01]L	XOR with $k_{p}$ profile to determine new profile of T conformation
UNUSED	4	[01]L	Reserved field

The asterisk means ‘zero or more’ and ‘+’ in regular expression means ‘one or more’.

#### Protein domain structure and allosteric flag

The coding sections of the binary string contain information about the domain structure of proteins (Figure 1A). Each protein must contain at least one domain that contains at least one reactive site. There is no maximum limit to the number of domains and reactive sites a protein can have. As explained above, domains may be allosteric units, and so, each domain is preceded with an allosteric flag sequence. When the allostery flag is set, the domain will undergo conformational changes and these dynamics may be affected by biochemical reactions happening at its reactive sites (note that reactions happening on other domains would not have an allosteric effect on this domain, i.e. domains are distinct and independent entities). To distinguish between domains and reactive sites on the binary string, we use ‘soft’ and ‘hard’ linker sequences that are inserted between domains and reactive sites respectively (Figure 1A). Thus, the ‘soft’ linker sequences indicate the start of a new domain within a protein; ‘hard’ linkers indicate the different reactive sites on a given domain whose conformational dynamics is potentially modulated by these sites (provided the domain is allosteric). This structure has the additional benefit that mutations that result in joining or separating of domains can be naturally implemented (see ‘Modeling Mutations’ section below). Reactive sites within a domain can be either a binding or catalytic site, and their nature is encoded on the binary string as shown in Table 1.

#### ANC intra-action fields

Intra-action fields are binary strings located at the beginning of each domain. They encode the parameters controlling the internal allosteric properties of the domain, namely the basal kinetic rates for the transitions between the R and T states ($k_{RT}$ and $k_{TR}$ from Equations [3] and [4]) and the parameter ${\rm \Phi }$ (which determines changes in allosteric equilibrium under interactions and is assumed to be the same for each of the different reactive sites of the domain and, as such, encoded once per domain). The switching rates are log linearly scaled into a real value (Figure 1F, Table 1).

**Table 2.** Parameter settings used for the *in silico* evolution of signaling networks discussed in the main text

Parameters	*In silico*	Measure	Citation
Cell volume (*pl*)	N/A	0.029, 1.2, 2.5	(92,93)
Nucleus volume (*pl*)	N/A	0.22, 0.14	(93)
Cytoplasmic volume (*pl*)	N/A	0.94, 2.4	(93)
Protein abundance	[0, 10^6^]	[38, 26743]	(92–94)
Concentration (*μM*)	[10^−3^, 10^3^]	[0.002, 1.8]	(12,92,93,95–98)
Phosphorylation ($s^{ - 1}$)	[10^−3^, 10^3^]	[0.17, 8.87]	(92,95–97)
Dephosphorylation ($s^{ - 1}$)	[10^−3^, 10^3^]	[0.06, 5.31]	(92,95–97)
Auto-dephosphorylation ($s^{ - 1}$)	N/A	[0.00097, 0.0025]	(92,95–97)
Binding receptors ($s^{ - 1}$)	[10^−3^, 10^3^]	[0.0036, 0.70]	(92,95–97)
Unbinding receptors ($s^{ - 1}$)	[10^−3^, 10^3^]	[0.00016, 0.060]	(92,95–97)
Protein association ($\mu M^{ - 1} \cdot s^{ - 1}$)	[10^−3^, 10^3^]	[0.10, 7.53]	(92,95–97)
Protein disassociation ($s^{ - 1}$)	[10^−3^, 10^3^]	[0.015, 2.86]	(92,95–97)
Conformation transition ($s^{ - 1}$)	[10^−2^, 10^2^]	N/A	N/A
Γ	[10^−2^, 10^2^]	N/A	(38)
Φ	[0.0, 0.1]	N/A	(38)

All of these parameters can be set by the user.

#### ANC interaction fields

Interaction fields are binary strings associated with the reaction sites in each domain. They encode how a change in the state of reaction site (binding or modification) will affect the R-T transition of that domain, i.e. they encode the parameters ${\rm \Gamma }_i$ described above. In addition, the binary string encodes binding and rate profiles (described in the next section), as well as a site type for each reactive site. The available types are *binding, catalytic* or *modification* sites (Table 1).

#### Binding and rate profiles

When the binary string is converted to an ANC model, BioJazz iterates over all pairs of reactive sites and compares their *binding profiles* to determine the site-specific interactions among protein domains. In each iteration, BioJazz performs an exclusive-OR (XOR) operation on the binding profiles of two given sites. The number of ‘1's in the string resulting from this operation determines whether or not binding occurs based on a user-defined threshold (Figure 1C). Besides the binding profile, each site has also a *forward* and *backward reaction rate profile*. When two sites are found to be binding (based on their binding profiles), the XOR operation is repeated, this time using the forward and backward rate profiles, to determine the binding coefficients (Figure 1E, Table 1). Finally, reactive sites that are catalytic encode an additional *catalytic rate profile*. If one of the sites is a catalytic site and the other a modification site, the catalytic rate profile of the former is scaled log linearly into a real value and is applied as the catalytic rate constant of the corresponding Michaelis–Menten kinetics. All the translated reaction rate constants are evolvable in biologically plausible parameter ranges (Table 2).

#### Profile masks

In real proteins, the kinetic rates associated with each reaction (e.g. binding rate, catalytic rate, etc.) can be altered by the structural changes that the protein undergoes. To include such changes, the model should incorporate the possibility of alterations in the kinetic rates of each reactive site with the R-T state transition of the domain. We do so by implementing a *conformational mask profile*, which is applied to all rate profiles of the reactive sites and alters the outcome of the XOR operation (Table 1). There are therefore distinct binding rates between the R and T states. For the modification sites only, there is also a *modification mask profile* that is applied to the binding rate profiles to alter the binding rates for modified states (Table 1). Note that both mask profiles can evolve to have no effect on kinetic rates, i.e. a given reactive site in a given domain can have the same reaction kinetic rates under each of the R and T states by appropriate setting of the mask profiles.

### Modeling mutations

The use of rule-based modeling and the encoding of such a model in a genome-like binary string allows us to implement most biologically feasible mutations in a natural way. Currently, the possible mutations included in BioJazz are point mutations, protein duplication, protein deletion, domain duplication, domain deletion, domain joining, domain splitting and domain shuffling. Of these, mutations involving domains were to our knowledge not considered before (17,31,54), but are straightforward to include in the rule-based approach. The rate of occurrence of the different mutations is controlled by user-defined parameters. Users can also restrict BioJazz to mutate a subset of the network's attributes including junk bits, linkers, binding profiles, allosteric flags, types of reactive site, *etc*. This flexibility is useful for example to ‘freeze’ all or parts of a network and use BioJazz as a design tool rather than mimicking biological evolution.

#### Point mutation

Point mutations are implemented as the flipping of specific bits in the binary string. Thus a point mutation can alter any of the qualitative flags (explained above) or reaction parameters. Of particular note are mutations on hard and soft linkers, which can result in domain splitting and fusion respectively. The mutation algorithm parses the binary string and attempts a point mutation at each location: a bit is flipped if a randomly generated number in the interval [0,1] is smaller than a user-set probability (corresponding to a genome-wide point mutation rate).

#### Protein duplication/deletion

In nature, the rate of gene duplication is suggested to be a function of the size of genome (55). Based on this observation, BioJazz implements duplication and deletion rates defined per protein. The mutation algorithm parses the binary string and attempts a duplication or deletion at each protein coding section; an entire section is duplicated or deleted if a randomly generated number in the interval [0,1] is smaller than a user-set probability. The protein duplication and deletion rates can be set independently. When a protein coding section is duplicated, it is added to the end of the binary string. When a protein coding section is deleted, the binary string is shortened correspondingly. It is also possible that a protein is silenced by a point mutation at its ‘start’ sequence.

#### Domain duplication/deletion

Bioinformatics analysis of the genomes of existing organisms reveals duplication patterns of domains in proteins, where the duplication patterns show no dependence on the size of the domains involved (56). Thus, BioJazz implements a per protein domain duplication/deletion rate. At each replication step, a randomly generated number in the interval [0,1] is generated for each protein. If this number is smaller than a user-defined probability, a random fragment of the binary string is picked. This fragment is then either deleted or copied and the new copy is inserted at the end of the original chosen fragment. Note that the randomly picked segment can contain many reactive sites or none.

#### Domain shuffling

BioJazz implements rearrangements between two protein-encoding sections of the binary string. The mutation rate leading to rearrangements is defined per protein. For each protein coding section of the binary string, a random number drawn from a uniform distribution in the interval [0,1] is compared to a user-defined probability. If the random number is smaller, a fragment containing a certain number of reactive sites is randomly chosen. Then, another subsection of a protein coding section of the binary string is randomly selected, copied and fused with the first selected fragment. Note that this approach combines sets of intact reactive sites, which can correspond to an entire domain, part of a domain or a sequence that covers multiple domains. Besides mimicking biological domain shuffling, shuffling is expected to create novel material for subsequent evolution.

#### Genome rearrangement

In biological systems, rearrangement of large genome chunks containing multiple genes also happens in certain probability. We also implemented this mutation operator in BioJazz. At each step of mutation, comparison between a random number and the rearrangement rate will determine occurrence of genome rearrangement. With rearrangement occurring, a continuous segment containing multiple reactive sites that possible cross several genes is randomly selected. Then either deletion of segment or duplication of segment is randomly chosen and executed.

#### Horizontal gene transfer

BioJazz also has implementation of horizontal gene transfer (HGT). Since HGT occurs between different genomes in nature, this mutational operator is only implemented when using population-based selection (see below). At the mutation step of each individual, the occurrence of HGT is determined by comparing a random number to a pre-defined probability (set by the user). If the random number is smaller than this probability, a continuous segment of string containing multiple reactive sites is randomly chosen and copied from the mutating individual. Then, another genome/individual is randomly selected and the copied segment is inserted into its genome at a randomly chosen site that is between any two reactive sites.

### Modeling evolutionary selective pressures

To simulate evolution *in silico*, we need to model selective pressures experienced by the evolving cellular networks and so link the contribution of a network's function to the overall *fitness* of the organism. Fitness is an abstract concept, representing the reproductive success of an organism and might be most tractable for microbes where it could be approximated by growth rate (57). In BioJazz, we allow the fitness of networks to be defined by the user, such that networks can be evolved under biologically motivated or artificial selective pressures.

The user-defined fitness function is used to evaluate the performance of a given network, encoded by a particular binary string and to calculate a fitness score. In previous studies on the evolution of signaling and regulatory networks, the fitness function usually involved applying a stimulus to the network and evaluating its temporal or steady state response (16,17,19,23,58,59). Different fitness functions relating to dynamical or structural features of the network can be easily constructed as illustrated in the results section for ultrasensitive (additional sample files are included in the BioJazz web site) and adaptive dynamics (see Figure 3 and Supplementary Data for a detailed description of the fitness functions)

**Figure 2.**
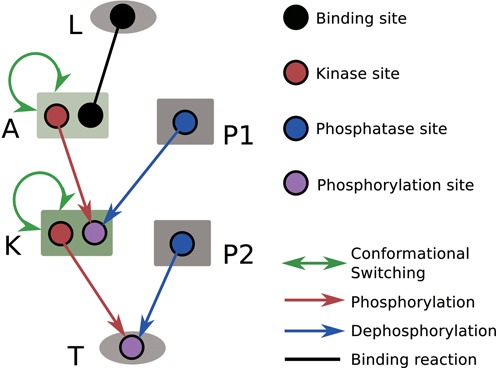
Schematic, showing the network structure used as the starting point for evolution for ultrasensitivity. The ligand (L) and the output protein (e.g. a transcription factor, T) are shaped as oval, while all other signaling proteins (e.g. a receptor/adaptor (A) protein, a kinase (K) or a phosphatase (P)) are shaped as rectangle. Black line represents binding reaction between two sites. Red arrows represent phosphorylation reactions between a kinase site (red) and a phosphorylation site (purple). Blue arrows represent dephosphorylation reactions between a phosphatase site (blue) and a phosphorylation site. The green colored rectangle indicates a protein domain, whose conformational switching is allosterically regulated (also indicated by a self-pointing green line with arrows at both ends).

**Figure 3.**
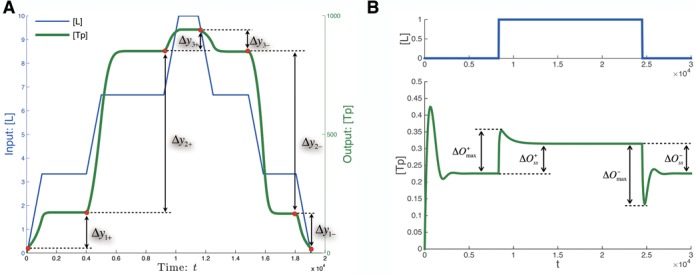
Sample fitness functions for selection for networks with ultrasensitive or adaptive response dynamics. (**A**) The input signal (blue) used in the temporal simulations of the system for ultrasensitivity. Each ramp-up and ramp-down of the signal is introduced after the system reaches steady state. The corresponding system output over time is shown in green. The differences in steady state output between different signal levels, indicated as Δ*y* values on the plot, are used to calculate the amplitude and ultrasensitivity scores. (**B**) Illustration of the dynamics of input signal (blue) the output response (green) in simulations of the system for adaptive dynamics. The parameters in adaptive fitness function, $\Delta O_{max}^{ + / - }$ and $\Delta O_{ss}^{ + / - }$, are labeled.

When the fitness function requires evaluation of the system dynamics, a temporal simulation of the network is executed by numerically integrating the set of ODEs arising from the interaction reactions in the network. To perform these simulations, Biojazz uses MATLAB with files automatically generated from ANC's output via the Facile tool (60). Stochastic simulation of the ANC model is also possible by customizing the fitness scoring function. Further, the flexibility we have in defining the fitness function means that fitness landscapes with different ruggedness as described for example for NK models of evolution (22,61–63) should be possible.

### Modeling evolutionary dynamics

Evolutionary dynamics arising from the emergence of mutant genotypes in a population and their subsequent change in frequency can be modeled in different ways. In particular, evolution could be approximated either by a random walk in which a single beneficial (or neutral) mutant can be fixed in the population before any other mutants can arise or as occurring in a population where multiple mutants can co-exist. The former is an appropriate model for evolutionary dynamics at low mutation rate and large population size limit (64,65); the latter approach can give rise to evolutionary dynamics similar to that described by the concept of quasi-species (66,67). Both approaches are implemented in BioJazz.

#### Evolution as a random walk

Under very low mutation rates and in large populations, evolutionary dynamics can be approximated by a random walk in the genotype space. Then, a single genotype dominates the population and new mutants either get fixed or are lost rapidly under natural selection and/or genetic drift (68). The probability of fixation for such rare mutants with a given fitness effect has been approximated by Kimura (64,65). This approximation can be used to model evolution under a large population and low mutation rate scenario, where the calculated probability of fixation for a mutant generated from the wild-type genotype is used to replace the wild-type or not (69,70). Biojazz implements this approach by starting simulations from a given genotype and using this genotype to generate a mutant genotype. The mutation is then accepted with probability $\alpha P_{fix}$, where $P_{fix}$ (fixation probability) is calculated from the fitness of the original (*w*) and mutant (*w’*) genotypes by the following equation:

(1) \begin{equation*} P_{fix} = \frac{{1 - e^{ - 2s} }}{{1 - e^{ - 4N_e s} }},\end{equation*}

with $s$ being the selection coefficient and equal to $s = \frac{{w\prime - w}}{w}$ and $N_e$ is the effective population size (set in the range $10^5 \sim 10^8$, based on measurements for prokaryotes (71)). The coefficient $\alpha$ is used to tune (usually increase) the speed of simulation and is always chosen to make $\alpha P_{fix} < 1$ for all mutations (69,70). A newly generated mutant will be accepted if a random number (uniformly drawn from the interval [0,1]) is smaller than $\alpha P_{fix}$. Otherwise it is rejected. After acceptance of a given mutant, that mutant replaces the original genotype and the simulation continues. If the mutant is rejected, a new mutant is generated from the original genotype. The evolutionary simulation is continued until a user defined fitness criterion or a specified number of mutations is reached.

#### Population-based approach

Here we consider evolution dynamics in discrete generations of an asexual population of a fixed-size (68,72). In a fixed-size population, selection for the next generation is implemented by sampling genotypes according to their fitness scores. Assume that there are genotypes $A_1,A_2,A_3, \ldots$ with fitness $w_1,w_2,w_3, \ldots$ and frequencies $p_1,p_2,p_3, \ldots$ in the current population. Then the expected proportion or frequency of $A_i$ genotypes in the next generation will be:

(2) \begin{equation*} p\prime _i = \frac{{p_i w_i }}{{p_1 w_1 + p_2 w_2 + \cdots }} = \frac{{p_i w_i }}{{\bar w}}.\end{equation*}

The $p_i^\prime$ is the propensity that genotype $A_i$ is chosen for reproduction (with one progeny) in each sampling. To implement these dynamics, we start with a homogenous population. At the beginning of each generation, individuals reproduce and mutate based on mutation rates by sequentially drawing and duplicating an individual and comparing the mutation rate with a random number $r_1$ from [0,1]. If $r_1$ is less than mutation rate, the reproduced individual is mutated. After reproduction the fitness scores for all mutants are recalculated. Then at the end of each generation, we apply selection. More specifically, we include all of the $p_i^\prime$ values in a vector and then generate another random number $r_2$ uniformly drawn between 0 and the length of this vector. The individual that is selected for the next generation is determined by the index of the vector into which the random number falls. The sampling process continues until the number of individuals in the new generation reaches the defined population size.

### BioJazz configuration file

BioJazz contains three key parts that are interlinked to each other: an encoding of an ANC model in the form of a binary string, evolutionary simulation of that binary string through mutations and dynamic simulation of the ANC model and derivation of a fitness score. Many of the parameters governing the structure of these three parts and their inter linkage can be defined by the user, allowing for high customizability. These parameters are stored in a single *configuration file*.

Besides the parameters already mentioned above, the configuration file also allows the setting of parameters relating to computational performance (e.g. cluster size, memory allocated for scoring), string encoding (e.g. fields’ width and binding profiles of input and output), the evolutionary algorithm (e.g. mutation rates, population size, seed network), the dynamical simulation of the ANC model (e.g. simulation time, numerical simulation error threshold), the scoring function (discussed below) and the output structure (e.g. frequency of output generation).

### Post-evolutionary pruning of evolved networks and mutational analysis

It is possible that not all reactions in the evolved networks are needed to achieve the required function (as seen for example in previous *in silico* evolution studies, (73,74)). Thus, we incorporated ways to either prune final networks or apply their mutations for further functional analyses. This can be done readily by altering the string representation of the network. BioJazz stores either each of the evolving networks (in the case of a population–based approach to modeling evolution) or the primary evolving network (in the case of the random walk approach to modeling evolution) at each generation of the simulation in two separate files. The user can choose to generate these files in either a BioJazz-compatible format or in additional formats readable in ANC, Facile and MATLAB. Pruning and mutations can be performed on these files and the resulting modified networks re-analyzed. When using BioJazz compatible files for such analysis, the user can make modifications to the string representation of the network and can also use existing subfunctions in the BioJazz source code. A detailed description and example of this approach is provided in the BioJazz manual.

## RESULTS

To illustrate the workings of BioJazz and how it can be used to address biological questions, we consider here the evolution of signaling networks under two example selective pressures (additional selective pressures can easily be constructed by encoding an appropriate fitness function in the configuration file, as shown in ‘Materials and Methods’ section) (Figure 2). Note that the fitness function used and the associated analyses are provided as an example to illustrate the applicability of BioJazz. The user has complete flexibility over the choice of fitness functions and of the parameters in a given evolutionary simulation.

Ultrasensitivity is observed in many biological networks and in particular in signaling networks implementing phosphorylation cycles (8,11,12,75). An ultrasensitive response is one where a change in the input generates a non-linear change in output with the degree of non-linearity being greater than that of a hyperbolic (Michaelis-Menten) response (8,75,76). To evolve signaling networks capable of displaying ultrasensitive dynamics, we run simulations with selection under a particular fitness function.

The fitness function used to score the ability of a given signaling network to generate an ultrasensitive signal-response relationship evaluated the response to a three-step ramp-up and three-step ramp-down signal profile as shown in Figure 3A. For each ramp-up step in the signal, the system is simulated to steady state before the next step is applied. The scoring function considered both the amplitude of the response to middle steps in ramp-up and ramp-down steps (amplitude score $S_{amp}$, see Supplementary Data) and the difference of the response amplitudes between the middle steps and the other two steps (ultrasensitivity score $S_{ult}$, see Supplementary Data). Besides the two scores quantifying the response and sigmoid, we also implemented a complexity score $S_{com}$ (Supplementary Data) to quantify the complexity of the network (i.e. total number of proteins, domains, reactive sites and interaction rules). The final fitness function combines the three scoring functions:

(3) \begin{equation*} F = \left( {S_{amp}^{\omega _a } \cdot S_{ult}^{\omega _u } \cdot S_{com}^{\omega _c } } \right)^{\frac{1}{{\left( {\omega _a + \omega _u + \omega _c } \right)}}} \end{equation*}

with the $\omega_{a}\mathrm{,}\ \omega_{u}\ \mathrm{and}\ \omega_{c}$ being user-defined parameters that control the weightings of the different scores.

With this fitness function, we used BioJazz to evolve ultrasensitive signaling networks. We started evolutionary simulations from a minimal seed network composed of a receptor, a kinase, a phosphatase and an output protein (Figure 2). The output protein was not allowed to duplicate or be deleted, but the rest of the network was free to evolve via all the mutations implemented in BioJazz (see ‘Materials and Methods’ section). Note that the constrained structure of the model in this case reflects a user choice rather than a limitation and allows us to demonstrate the application of BioJazz to evolve signaling networks with ultrasensitive dynamics by fixing the input and output of the evolving system. We could include the ligand as part of the evolving entity, in which case we would be able to evolve new ligands and ligand–receptor interactions, provided that an appropriate fitness function is devised. For example, to study the coevolution between ligands and the response, one can easily cluster different proteins based on the tags and prefixes of protein names implemented in the source code.

Selecting for ultrasensitivity in the signaling network using the random-walk approach (see ‘Materials and Methods’ section), we ran evolutionary simulations by assuming a high population size and low mutation rate regime (see ‘Materials and Methods’ section) and by using different complexity weightings $\omega _c$. In particular, we ran five simulations each for four different complexity weights: $\omega _c = 0,0.1,1,10$. We set a target fitness score of 0.8 and a maximal computation time of 120 h per simulation. The simulations were terminated when either the target fitness score or simulation time was reached.

**Figure 4.**
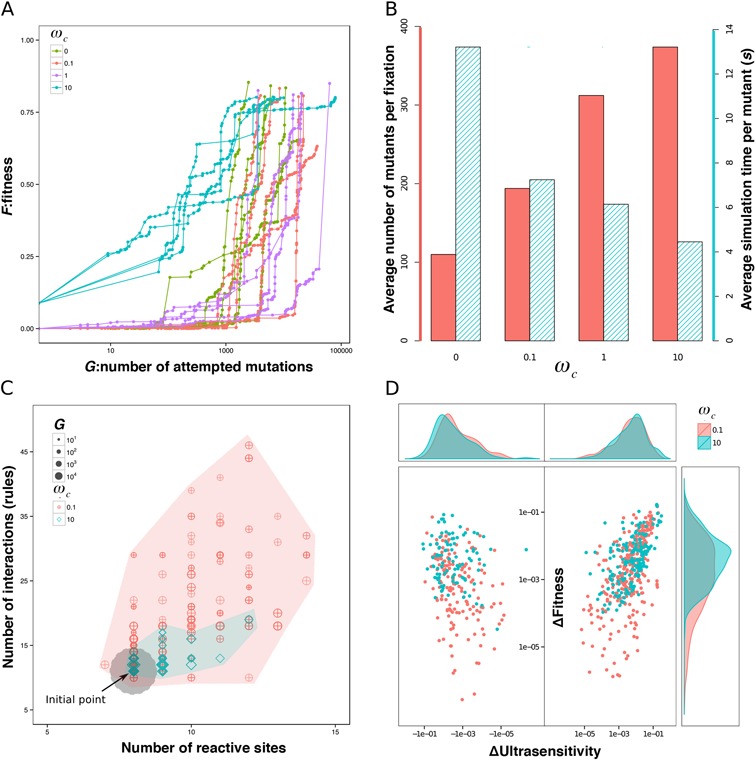
Results from sample evolutionary simulations. (**A**) The fitness score plotted against the total number of mutations sampled. Each curve depicts the results of a single evolutionary simulation, which is a biased random walk over the network space (Equation [5] in ‘Materials and Methods’ section). Each dot on each curve represents an accepted mutation (lines are to guide the eye). Distances along the x-axis between two dots indicate the number of mutations sampled between two accepted mutations. In all simulations, fitness increases with the number of mutations accepted, but in two simulations (one with $\omega _c = 0$ and one with $\omega _c = 0.1$) fitness fails to reach the target level of 0.8 before the maximal simulation time of 120 h is reached. (**B**) The average number of mutants sampled before a mutation is accepted increases with $\omega _c$; the average time for evaluating the fitness of each mutant in simulations decreases with $\omega _c$. A higher weighting of complexity score ($\omega _c$) in the total score gives a higher penalty to mutations that generate complexity in the network structure. (**C**) The evolutionary space showing the numbers of reactive sites and of interactions for all simulations with $\omega _c = 0.1{\rm and}10$. Each data point represents an accepted mutant network from different stages of the simulation, with the shape and color indicating the $\omega _c$ of the simulation and the size indicating the generation number (i.e. number of mutations). Note that many of the data points from the simulations with $\omega _c = 10$ are overlapping. The initial network is at the center of the gray area. (**D**) The distributions of mutational effects on fitness and ultrasensitivity from accepted mutations during all simulations with $\omega _c = 0.1{\rm and}10$ (as indicated in red and green respectively). Sub-graphs at the top and right are density estimates for the ultrasensitivity changes $\Delta S_{ult}$ and fitness changes $\Delta F$ of all fixation events.

In all simulations, the fitness score increases over generations (Figure 4A) and we evolve an ultrasensitive network reaching at least a fitness score of 0.8. Analyzing the evolutionary dynamics in these simulations, we find that fewer mutations were needed for simulations with $\omega _c$ set to lower values (Figure 4B), i.e. when the fitness penalty for complexity was low. The time required for evaluating the fitness of each mutant, however, was significantly larger with lower $\omega _c$. These findings suggest that a weaker constraint on network complexity (i.e. smaller values of $\omega _c$) allows the evolutionary simulations to sample a larger space of networks and more easily find beneficial mutants. Correspondingly, the number of reactive sites and interactions in networks diverges more widely in such simulations, while network complexity is highly constrained for large $\omega _c$ (Figure 4C). On the other hand, a higher weighting for the complexity measure (high $\omega _c$) can result in this measure dominating the total fitness calculation (Equation 3). Consequently, a larger number of mutations with detrimental or neutral effects on the ultrasensitivity and amplitude of the response may be accepted because their low scores could be absorbed by stronger effects from the complexity measure. We find that indeed this possibility is realized: the distribution of the ultrasensitivity scores of fixed mutants is slightly shifted to larger negative values in simulations with $\omega _c = 0.1$ compared to data from simulations with $\omega _c = 10$ (Figure 4D). A similar pattern also occurs with amplitude.

**Figure 5.**
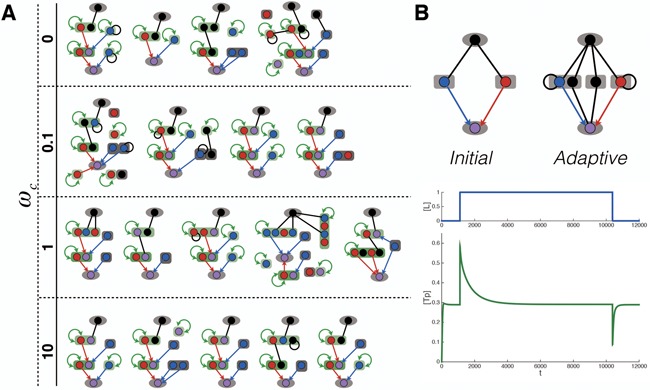
Sample evolved network structures and dynamics. (**A**) Sample network structures evolved to achieve ultrasensitivity in simulations with different weighting of the complexity score $\omega _c$. In each network, the nodes stand for proteins and edges stand for interactions. The isolated (i.e. unconnected) nodes seen on some of the evolved networks represent proteins that do not interact with any other proteins (hence they can be removed without affecting the response dynamics). For explanation of labels and edge colors see legend of Figure 2. (**B**) An evolved network structure and its dynamics using selection for an adaptive response.

**Figure 6.**
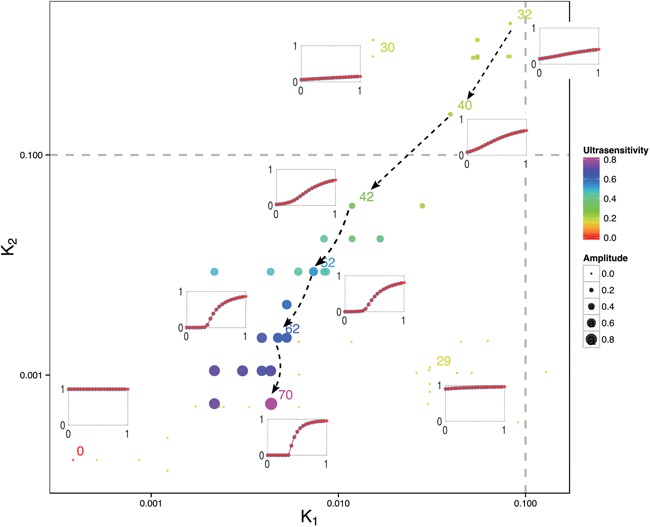
Evolution of model parameters for a sample evolutionary simulation with $\omega _c = 0$ and selecting for ultrasensitivity. Each dot represents a model from different points in the evolutionary simulation (as indicated by the generation number on each dot), while the x- and y-axis show the composite parameters, $K_1$ and $K_2$, that give the average catalytic binding efficiency of the kinases and phosphatases to the target protein respectively. The catalytic binding efficiency is defined as the Michaelis Menten constant of the enzyme (kinase or phosphatase) over the total substrate concentration and the average is calculated as the geometric mean of individual binding efficiencies of the different kinases and phosphatases and their allosteric states: $K_1 = \mathop \prod \limits_{i = 1}^m \sqrt[m]{{(K_1^{R_i } \cdot K_1^{T_i } )^{\frac{1}{2}} }}\;{\rm and}\;K_2 = \mathop \prod \limits_{j = 1}^n \sqrt[n]{{\left( {K_2^{R_j } \cdot K_2^{T_j } } \right)^{\frac{1}{2}} }}$. The dot size and color indicate the response amplitude and ultrasensitivity. For selected networks the input-output response curve is also shown. Dashed lines with arrow heads show the trend of how ultrasensitivity increases with the evolution of decreasing values of $K_1 {\rm and}K_2$. The initial network starts in the bottom left corner (network 0) moves to the bottom right corner (network 29) and then to the top right corner (network 32).

Our implementation of evolution under a low mutation rate and high population size regime through Equation [5] still allows for a degree of neutral evolution. Thus, we find significant diversity in the set of ultrasensitive networks emerging at the end points of different evolutionary simulations (Figure 5A). This diversity confirms that different network architectures and biochemical mechanisms can generate ultrasensitivity. The evolved ultrasensitive networks we find recover known biochemical mechanisms that generate ultrasensitivity. One such mechanism is enzyme saturation in a covalent modification cycle (or zero-order sensitivity or Goldbeter–Koshland kinetics) (77). In this mechanism, saturation of enzymes that mediate the covalent modification of a substrate generates ultrasensitivity in the modified substrate levels. In our simulations, the initial starting networks display high levels of kinase and phosphatase and low levels of target protein, and we analyzed the evolutionary trajectory of key kinetic parameters in a few sample simulations. In particular, we consider composite parameters $K_1 {\rm and}K_2$, which determine the binding kinetics of the kinase and phosphatase to the output protein and should decrease with increased enzyme saturation (see the legend of Figure 6 for a full definition of $K_1 {\rm and}K_2$). We find that the initial evolution of these parameters is quite erratic (Figure 6) until the system reaches a high level of $K_2$ where phosphorylation can result in low output at any signal level (network 30). Once this point is reached, evolution progresses with both $K_1 {\rm and}K_2$ being decreased, indicating that the enzymes (kinase and phosphates) spend less time in complexes with the output protein: the enzymes increasingly become saturated. Consequently, both the ultrasensitivity and the amplitude of the system response increase and reach the target fitness score in network 70, whose MATLAB code is also provided as additional Supplementary Data files. We find a similar trend in other simulations, where decreasing $K_1 {\rm and}K_2$ is accompanied by increasing ultrasensitivity, suggesting that these trajectories may be common in the evolution of ultrasensitive responses, at least from an initial regime of high substrate and low enzyme concentration.

To provide a second example for the application of BioJazz, we developed a different fitness function that is designed to select for networks with adaptive response dynamics (Figure 3B, see Supplementary Data). This type of response dynamics is observed in many cellular systems and is characterized by an initial response to a persistent external stimulus that eventually returns to its pre-stimulus level. In the context of signaling networks, adaptive response dynamics are observed and studied in bacterial chemotaxis (78–80) and the response of yeast to osmotic shock (81–83). General signaling network models capable of adaptation have been presented (84) and *in silico* evolution has been successfully used to understand gene network architectures that can achieve adaptive responses (17). Here, we have adopted the fitness function used in the latter study (see Supplementary Data) and used BioJazz to evolve signaling networks with adaptive dynamics (Figure 5B). We found that 9 out of 10 from the initiated simulations resulted in networks achieving high fitness solutions and adaptive response dynamics. Different from previous work on adaptive gene networks, the structures of evolved adaptive protein interaction networks do not show any obvious negative feedback (4). Instead, we find the evolved networks commonly exploiting a buffering mechanism that could be equivalent to a feedforward mechanism (4). In the example adaptive network shown in Figure 5B, the input protein can bind four binding sites in three different proteins, two of which are the kinase and phosphatase for the output protein. When a perturbation happens at the input protein concentration level, different affinities of kinase and phosphatase for binding to the input protein result in breaking the balance of phosphorylation and dephosphorylation of the output protein, inducing an initial response. Later, the binding protein in the middle (which has slower binding reaction rate constants) sequesters the input protein to rebalance the phosphorylation and dephosphorylation of the output protein. The end effect of this buffering mechanism is a response dynamics similar to that resulting from a feedforward interaction loop (4). All other evolved adaptive networks utilized similar solutions to this example to achieve adaptive responses. Analyzing the dynamics of sample evolved networks under different levels of input perturbation we found their fitness to be sensitive to the level of the perturbation used in the fitness function. In particular, the adaptation precision (i.e. the ability to return exactly to pre-stimulus activity level after a signal) is dependent on signal level. This highlights the importance of the design of the fitness function on the types of networks that can evolve in the simulations. In future studies BioJazz can be used to evolve adaptive networks under different fitness functions (e.g. those with varying signal perturbation levels) to better understand the design principles for adaptation in protein interaction networks.

## DISCUSSION

Here we have presented BioJazz, a tool that combines rule-based approaches and evolutionary simulation. Its key features are the implementation of biochemical interactions found in cellular networks, the simulation of dynamics arising from these interactions and their evolution with unbounded complexity through biologically plausible mutations. Previous approaches to evolutionary simulation of cellular networks have only considered a subset of these abilities. As such, we expect BioJazz to be useful both as an exploratory tool for the evolutionary systems biology community to understand evolutionary pressures leading to specific biochemical features of biological networks and as a design tool for the synthetic biology community to explore biochemically plausible implementations of different network dynamics.

As we demonstrate, BioJazz is developed in a way that allows high flexibility and user-friendliness. All parameters relating to the evolutionary simulations, as well as the fitness functions used to select networks can be specified by the user, allowing testing of different hypotheses. As a demonstration, we showed how to use BioJazz to evolve networks under different complexity constraints and to generate ultrasensitive dynamics. We found that complexity constraints can alter the efficiency of the evolutionary simulations, mainly because of their effects on the distribution of mutational effects on fitness.

Under all complexity constraints considered, we found evolutionary simulations to result in ultrasensitive networks under the appropriate fitness function. In addition, adoption of a different fitness function allowed the evolution of networks displaying adaptive dynamics. These results show that BioJazz can be used to study a range of system dynamics (i.e. ultrasensitivity, adaptation, oscillation). Networks resulting from specific simulations that implemented different selective pressures displayed specific architectures, suggesting that BioJazz can be used to study the possible repertoire of functional networks. In the case of ultrasensitivity, we found that these networks and their evolutionary dynamics highlighted known biochemical mechanisms. In particular, we found that kinetic parameters controlling binding of the enzymes and output protein evolve to favor low saturation initially for increased response amplitude and then high saturation later on for increased ultrasensitivity. BioJazz can be used to further elucidate such trends under different evolutionary scenarios. For example, the simulations we used started from high substrate and low enzyme concentrations. It would be interesting to reverse this situation and explore how ultrasensitivity can emerge under regimes where high enzyme saturation would not be possible (9). Similarly, one can use higher level selection functions, rather than *ad hoc* functions selecting for ultrasensitivity (as we have done here), to elucidate the biological origins of ultrasensitivity. Alternatively, one can implement selection for different dynamics such as adaptive response dynamics or oscillatory dynamics. The evolved network structures could then provide insights into which biochemical networks can implement the required dynamics and inform both systems and synthetic biology studies (as has been done before, e.g. see (23,24,31,32)).

There are notable previous works on evolutionary simulation of the structure and dynamics of cellular networks. In particular, previous studies analyzed the *in silico* evolution of gene regulatory networks to understand the emergence of different dynamics (16,17,35,73,85) and their modularity and robustness (86). The latter features were also studied in evolutionary simulations using either metabolic (87) and signaling network models (54,74,88) or general network models (26,27). As an open-source platform, BioJazz aims to further enable such studies by providing an *in silico* evolution model that explicitly considers systems dynamics and protein allostery and domain structure. The incorporation of protein allostery and domain structure is a unique addition to the evolutionary modeling of networks, but whose effects on system dynamics have been demonstrated (36,37). In addition, the combination of rule-based modeling with *in silico* evolution is a novel approach to modeling evolution and naturally includes emerging system complexity in evolutionary simulations. In particular, the rule-based modeling approach theoretically allows for simulation of arbitrarily large reaction networks as well as protein complexes.

Although by using rule-based modeling BioJazz theoretically allows the evolution of cellular networks without restricting their complexity, there are still computational challenges when simulating large reaction networks and multi-protein complexes that give rise to the ‘curse of dimensionality’ (38,40). In particular, the ANC framework used here generates the full set of differential equations possible in the network, prior to simulation, which can create a significant computational burden. Such technical challenges are increasingly being addressed with developing rule-based modeling frameworks. For example, the Kappa simulator KaSim (89) and the BioNetGen simulator NFsim (44,90) both allow faster simulation of reaction systems of arbitrary size. These methods are currently based on using stochastic simulations and do not consider the allosteric nature of proteins as done in ANC. It should be possible to combine the best features of the developing approaches and create new rule-based modeling frameworks that combine modeling of protein allostery with computationally feasible simulation methods allowing arbitrarily large networks to be simulated. Future development of BioJazz will expand the rule-based modeling aspect of its evolutionary framework towards combining the best features of different methods.

Such development of the rule-based modeling component of BioJazz would extend its focus from encoding signaling networks to include metabolic and transcriptional networks. In particular, rule-based models like Kappa and BioNetGen are able to model degradation and synthesis reactions. This ability can be combined with the binary string patterns of a BioJazz model to encode binding between proteins and genes, and thereby simulate transcription factors binding to DNA. For metabolic networks, the extension would require encoding of metabolites in a form that captures the basics of chemical conversion (87,91). This extension would require significant further development and interfacing rule-based models and metabolites through their corresponding representations and with biological plausible parameters (12,38,92–98) (Table 2). We hope that such developments will be facilitated by the open-source nature of BioJazz.

## SUPPLEMENTARY DATA

Supplementary Data are available at NAR Online.

SUPPLEMENTARY DATA
