# Supplementary material for: BioJazz: in silico evolution of cellular networks with unbounded complexity using rule-based modeling
Source: Nucleic Acids Res. 2015 Jun 22;43(19):e123. doi: 10.1093/nar/gkv595 (PMC4627059; doi:10.1093/nar/gkv595)
Supplement: SUPPLEMENTARY DATA [file supp_43_19_e123__index.html]

BioJazz: in silico evolution of cellular networks with unbounded complexity using rule-based modeling — BioJazz: in silico evolution of cellular networks with unbounded complexity using rule-based modeling — SUPPLEMENTARY DATA 

# BioJazz: *in silico* evolution of cellular networks with unbounded complexity using rule-based modeling

## SUPPLEMENTARY DATA

- SUPPLEMENTARY DATA
- SUPPLEMENTARY DATA
