# Supplementary material for: BioJazz: in silico evolution of cellular networks with unbounded complexity using rule-based modeling
Source: Nucleic Acids Res. 2015 Jun 22;43(19):e123. doi: 10.1093/nar/gkv595 (PMC4627059; doi:10.1093/nar/gkv595)
Supplement: SUPPLEMENTARY DATA [file supp_gkv595_nar-00412-met-z-2015-File008.docx]

**Supplementary Document to “BioJazz: *in silico* evolution of cellular networks with unbounded complexity using rule-based modeling”**

**Song Feng^1,&^, Julien F Ollivier^2,&^, Peter S Swain^3,*^, and Orkun S Soyer^1,*^**

^*^Corresponding authors. ^&^These authors contributed equally to this work.

**1. The energy framework implemented in ANC for modelling allosteric regulation**

In particular, the Arrhenius equation gives the kinetic rate of the R-T transition as: $k_{RT}=k_{R\dagger}=Ae^{-\Delta G_{R\dagger}/kT}$, with “$\dagger$” denoting the transition state, *A* denoting the Arrhenius constant, and $kT$ being the product of Boltzmann’s constant and temperature {DePaula 2006}. Similarly, $k_{TR}=k_{T\dagger}=Ae^{-\Delta G_{T\dagger}/kT}$. The equilibrium distribution of the R and T states will be governed by the equilibrium constant $K_{RT}$, which is given by ${k_{RT}}/{k_{TR}}$, where $K_{RT}=Ae^{{(-\Delta G_{R\dagger}+\Delta G_{T\dagger})}/{kT}}=Ae^{{-\Delta G_{RT}}/{kT}}$.

In ANC, modifiers are assumed to contribute independently to the free energy of each conformational state, R and T, allowing us to formulate the free energy difference between these two states ($\Delta G_{RT}^{'}$) in a given domain with $N$ modifiers as:

| $\boldsymbol{\Delta}\boldsymbol{G}_{\boldsymbol{RT}}^{\boldsymbol{'}}\boldsymbol{= \Delta}\boldsymbol{G}_{\boldsymbol{RT}}\boldsymbol{+}\sum_{\boldsymbol{i=1}}^{\boldsymbol{N}} \left( \boldsymbol{\Delta}\boldsymbol{G}_{\boldsymbol{T}}^{\boldsymbol{(i)}}\boldsymbol{-\Delta}\boldsymbol{G}_{\boldsymbol{R}}^{\boldsymbol{(i)}} \right)\boldsymbol{,}$ |  | (1) |
| --- | --- | --- |

where $\Delta G_{T}^{(i)}$ and $\Delta G_{R}^{(i)}$ give the effect of the $i^{th}$ modifier on the free energies of the R and T states. While $\Delta G_{RT}^{'}$ could be evaluated via equation [1], this requires assignment of the $\Delta G_{T}^{(i)}$ and $\Delta G_{R}^{(i)}$ values. Instead of doing this, we can exponentiate [1] and thus equivalently define the effect of each modifier on the overall equilibrium distribution between the R and T states. To do so, we defined the relation of the equilibrium constant of the domain without any modifiers ($K_{RT}$) to that with modifiers ($K_{RT}^{'}$) as:

| $\frac{\boldsymbol{k}_{\boldsymbol{RT}}^{\boldsymbol{'}}}{\boldsymbol{k}_{\boldsymbol{TR}}^{\boldsymbol{'}}}\boldsymbol{=}\boldsymbol{K}_{\boldsymbol{RT}}^{\boldsymbol{'}}\boldsymbol{=}\boldsymbol{K}_{\boldsymbol{RT}}\prod_{\boldsymbol{i=1}}^{\boldsymbol{N}} \boldsymbol{\Gamma}_{\boldsymbol{i}}\boldsymbol{,}$ | (2) |
| --- | --- |

where $\Gamma_{i}=e^{-(\Delta G_{T}^{\left( i \right)}-\Delta G_{R}^{\left( i \right)})/kT}$ denotes the effect of the $i^{th}$ modifier on the equilibrium distribution between the R and T states. The $\Gamma_{i}$ relate to the altered kinetic rate constants in the presence of the $i^{th}$ modifier in the following manner:

| $\boldsymbol{k}_{\boldsymbol{RT}}^{\boldsymbol{'}}\boldsymbol{=}\boldsymbol{k}_{\boldsymbol{RT}}\prod_{\boldsymbol{i=1}}^{\boldsymbol{N}} \left( \boldsymbol{\Gamma}_{\boldsymbol{i}} \right)^{\boldsymbol{\Phi}_{\boldsymbol{i}}}\boldsymbol{,}$ | (3) |
| --- | --- |

| $\boldsymbol{k}_{\boldsymbol{TR}}^{\boldsymbol{'}}\boldsymbol{=}\boldsymbol{k}_{\boldsymbol{TR}}\prod_{\boldsymbol{i=1}}^{\boldsymbol{N}} \left( \boldsymbol{\Gamma}_{\boldsymbol{i}} \right)^{\boldsymbol{(\Phi}_{\boldsymbol{i}}\boldsymbol{-1)}}\boldsymbol{,}$ | (4) |
| --- | --- |

with the parameter $\Phi_{i}$ describing the proportional effects of the $i^{th}$ modifier on the R-T transitions. To simplify the implementation of this approach, all modifiers acting on different reactive sites of a domain are assumed to employ the same $\Phi$ value (i.e. $\Phi_{i}=\Phi_{j} (i\neq j)$ for all reactive sites in one domain) {Ollivier 2010}.

**2. Calculation of different scores in ultresensitivity fitness function**

If we define$y_{min}$ and $y_{max}$ as the minimum and maximum values of the response during the interval from a change in the signal to steady-state, then the response amplitude for each of the signal ramp-ups (indicated with a ‘+’ sign) and ramp-downs (indicated with a ‘-’ sign) is calculated as:

| $\boldsymbol{\Delta}\boldsymbol{y}_{\boldsymbol{i+}}\boldsymbol{=}\boldsymbol{y}_{\boldsymbol{i+}_{\boldsymbol{max}}}\boldsymbol{-}\boldsymbol{y}_{\boldsymbol{i+}_{\boldsymbol{min}}}$ | (5) | $\boldsymbol{\Delta}\boldsymbol{y}_{\boldsymbol{i-}}\boldsymbol{=}\boldsymbol{y}_{\boldsymbol{i-}_{\boldsymbol{max}}}\boldsymbol{-}\boldsymbol{y}_{\boldsymbol{i-}_{\boldsymbol{min}}}$ | (6) |
| --- | --- | --- | --- |

where the subscripts denote the the corresponding ramps in the input signal. With these measurements, the amplitude score ($S_{amp}$) is given as the normalized amplitude of the change in response to the second ramp-up and ramp-down signals:

| $\boldsymbol{S}_{\boldsymbol{amp}}\boldsymbol{=}\frac{\boldsymbol{(\Delta}\boldsymbol{y}_{\boldsymbol{2+}}\boldsymbol{+\Delta}\boldsymbol{y}_{\boldsymbol{2-}}\boldsymbol{)/2}}{\boldsymbol{y}_{\boldsymbol{total}}}$ | (7) |
| --- | --- |

with $y_{total}$ being the maximum possible response (i.e. the concentration difference between a fully active and fully inactive output protein), and acts as a normalization factor ensuring $S_{amp}$ to be between 0 and 1.

To quantify the ultrasensitivity of the system, we use the difference between the amplitudes of the responses to the second ramp-up/ramp-down signals, and the first choice/third choice. We first define the difference in the response to the different ramp-up and ramp-down signals as:

| $\boldsymbol{S}_{\boldsymbol{u}\boldsymbol{1}}\boldsymbol{=}\frac{\boldsymbol{(\Delta}\boldsymbol{y}_{\boldsymbol{2+}}\boldsymbol{+\Delta}\boldsymbol{y}_{\boldsymbol{2-}}\boldsymbol{)/2}}{\boldsymbol{(\Delta}\boldsymbol{y}_{\boldsymbol{1+}}\boldsymbol{+\Delta}\boldsymbol{y}_{\boldsymbol{1-}}\boldsymbol{)/2}}$ | (8) | $\boldsymbol{S}_{\boldsymbol{u}\boldsymbol{3}}\boldsymbol{=}\frac{\boldsymbol{(\Delta}\boldsymbol{y}_{\boldsymbol{2+}}\boldsymbol{+\Delta}\boldsymbol{y}_{\boldsymbol{2-}}\boldsymbol{)/2}}{\boldsymbol{(\Delta}\boldsymbol{y}_{\boldsymbol{3+}}\boldsymbol{+\Delta}\boldsymbol{y}_{\boldsymbol{3-}}\boldsymbol{)/2}}$ | (9) |
| --- | --- | --- | --- |

and the ultrasensitivity score ($S_{ult}$) is then calculated as:

| $\boldsymbol{S}_{\boldsymbol{ult}}\boldsymbol{=}\sqrt{\left( \frac{\boldsymbol{S}_{\boldsymbol{u}\boldsymbol{1}}}{\boldsymbol{r}_{\boldsymbol{u}}\boldsymbol{+}\boldsymbol{S}_{\boldsymbol{u}\boldsymbol{1}}} \right)\boldsymbol{\cdot}\left( \frac{\boldsymbol{S}_{\boldsymbol{u}\boldsymbol{3}}}{\boldsymbol{r}_{\boldsymbol{u}}\boldsymbol{+}\boldsymbol{S}_{\boldsymbol{u}\boldsymbol{3}}} \right)}$ | (10) |
| --- | --- |

In equation [12], $r_{u}$ is a user-defined scaling parameter that ensures the two ratios $S_{u1}$ and $S_{u3}$ (and thus the ultrasensitivity score) is between 0 and 1.

Besides the amplitude and ultrasensitivity scores, we also define a complexity score ($S_{com}$). It is plausible to assume that networks are under selection to minimize their energetic burden to the cell, and this score allows us to capture network complexity. The complexity score is given by;

| $\boldsymbol{S}_{\boldsymbol{com}}\boldsymbol{=}\frac{\boldsymbol{r}_{\boldsymbol{c}}}{\boldsymbol{r}_{\boldsymbol{c}}\boldsymbol{+C}}$ | (11) |
| --- | --- |

where $C$ is the sum of the total number of rules, proteins, domains, and reactive sites in the ANC model and $r_{c}$ is a user-defined scaling parameter for scaling the complexity score $S_{com}$ between 0 and 1.

**3. Calculation of fitness function quantifying adaptive response**

The key aspects of adaptive response dynamics are that the system shows an initial response to the input ($\Delta O_{max}^{+/-}$ ≠ 0) and that the steady state value of the output returns to its pre-input level, irrespective of the level of the input. In other words, after a sustained change in input (e.g. a step change), the output should initially respond but ultimately settle back to its original steady state: $\Delta O_{ss}^{+/-}\approx0$. Therefore, the adaptation fitness $w$ can be configured as:

| $w=\sqrt{\frac{\Delta O_{max}^{+}}{C}\times\frac{K}{K+\Delta O_{ss}^{+}}}\cdot\sqrt{\frac{\Delta O_{max}^{-}}{C}\times\frac{K}{K+\Delta O_{ss}^{-}}}$ | (12) |
| --- | --- |

where $C$ is a normalization factor to scale $\Delta O_{max}^{+/-}$ and $\Delta O_{ss}^{+/-}$ in to $[0,1]$, and $K$ is a threshold parameter. By imposing such a selective pressure, it is possible to evolve an increased response sensitivity ($\Delta O_{max}^{+/-}$) and reduced adaptive error ($\Delta O_{ss}^{+/-}$), and so achieve networks with an adaptive response.
